# Enhanced trimethylamine metabolism and gut dysbiosis in type 2 diabetes mellitus with microalbumin

**DOI:** 10.3389/fendo.2023.1257457

**Published:** 2023-11-20

**Authors:** Lixia Huo, Hui Li, Ming Zhu, Yang Liu, Lingyan Ren, Jia Hu, Xiaoyi Wang

**Affiliations:** ^1^ Huzhou Key Laboratory of Translational Medicine, The First Affiliated Hospital of Huzhou University, The First People’s Hospital, Huzhou, Zhejiang, China; ^2^ Department of Environmental and Occupational Health, Center for Disease Control and Prevention, Huzhou, Zhejiang, China; ^3^ Department of Nephrology, The First Affiliated Hospital of Huzhou University, The First People’s Hospital, Huzhou, Zhejiang, China; ^4^ Department of Endocrinology, The First Affiliated Hospital of Huzhou University, The First People’s Hospital, Huzhou, Zhejiang, China

**Keywords:** type 2 diabetes mellitus with microalbuminuria, gut microbiota, trimethylamine, trimethylamine-n-oxide, urinary albumin creatinine ratio

## Abstract

**Background:**

Abnormal gut microbiota and blood trimethylamine-N-oxide (TMAO) metabolome have been reported in patients with type 2 diabetes mellitus (T2DM) and advanced diabetic nephropathy. This study aimed to investigate the gut microbiota profiles and a group of targeted urine metabolic characteristics in T2DM patients with or without microalbuminuria, to determine the correlation between the gut microbiota composition, trimethylamine (TMA) metabolism, and the clinical features during progression of diabetic kidney disease (DKD)

**Methods:**

This study included 26 T2DM patients with microalbuminuria (Micro), 26 T2DM patients with normoalbuminuria (Normo), and 15 healthy controls (HC). Urine and Fecal samples were detected using ultra performance liquid chromatography tandem mass spectrometry and 16S ribosomal DNA gene sequencing, respectively.

**Results:**

The TMAO/TMA ratio decreased gradually during the HC-Normo-Micro transition. The levels of TMA, choline and betaine were significantly different between the HC group and the T2DM patients belonging to both Normo and Micro groups. At the operational taxonomic unit (OTU) level, the gut microflora diversity was significantly reduced in the Micro groups compared to the HC groups and the Normo groups. Taxonomic analyses revealed significant consumption in the relative abundances of eight bacterial genera and significant enrichment of two bacterial genera during the HC-Normo-Micro transition. Furthermore, the relative abundances of six bacterial genera, namely, Ruminococcus_1, [Eubacterium]_ruminantium_group, Roseburia, Faecalibacterium, Fusicatenibacter and Coprococcus_3 exhibited significant differences, and were associated with elevated urinary albumin creatinine ratio (UACR), TMAO/TMA, TMA and its precursors in the Micro group compared with the other groups.

**Conclusion:**

The imbalance of gut microbiota has occurred in patients with early-stage DKD, and the consumption of short-chain fatty acid-producing bacteria were associated with the accumulation of TMA and UACR.

## 1 Introduction

Diabetic kidney disease (DKD) is the most concerning microvascular complication of diabetes mellitus (DM) and a leading cause of end-stage renal disease (ESRD) and cardiovascular disease (CVD) (1, 2). Up to 40% of type 2 diabetes mellitus(T2DM) patients develop to DKD, and the all-cause mortality is significantly higher in DKD patients compared to DM patients without kidney disease (3). DKD patients demonstrate a higher risk of developing CVD compared to the risk of the disease progressing to ESKD (4). The early detection of DKD is limited, and most of the DKD patients are diagnosed at advanced stages III or IV, which is largely irreversible (5). Current approaches, including lifestyle changes, management of glycemic and blood pressure control, provide limited effects in reducing the incidence and progression of DKD. Therefore, identifying early causes of DKD and developing intervention strategies are of great significance.

The gut microbiota has gained increasing attention as a significant environmental factor in various chronic diseases, including obesity, DM, CVD, nonalcoholic fatty liver disease and chronic kidney disease (CKD) (6, 7). Recent research has revealed differences in the gut microbiota composition between DKD patients and healthy individuals (8, 9). However, most of these studies have focused on the mid to late stages of DKD, with limited investigations in the early stages.

Recent studies have reported that the gut microbiota-derived metabolites play a significant role in disease development by interacting with the host via multiple pathways. Trimethylamine (TMA) is a crucial gut microbiota-derived metabolite that is primarily converted to trimethylamine-N-oxide (TMAO) by the liver enzyme flavin-containing monooxygenase 3. Approximately 95% of TMAO is eliminated by the kidneys. Furthermore, accumulation of TMAO is associated with renal dysfunction. In CKD, elevated levels of TMAO are associated with renal insufficiency and increased risk of mortality (10, 11). Higher TMAO levels are associated with decreased glomerular filtration rate and impaired renal function. Gut dysbiosis aggravated renal dysfunction by increasing the levels of toxic metabolites in the blood (12). TMAO levels are elevated in cases of gut dysbiosis. Haluzik et al. (13) reported that alterations in the intestinal flora of patients with CKD resulted in an increased ability to metabolize choline and elevated transcription of choline monooxygenase and other genes, which increase the levels of TMAO; furthermore, elevated TMAO levels were associated with poorer prognosis of patients with DKD. Higher TMAO levels are associated with increased inflammation (14), abnormal renal function, and renal fibrosis. TMAO promoted renal interstitial fibrosis through the TGF-β/Smad3 signaling pathway. Moreover, TMAO levels and the TMA/TMAO ratio are independent risk factors of DKD (15). T2DM patients with advanced CKD showed increased abundance of TMA-producing bacteria and elevated serum TMAO levels (16). However, very few studies have investigated the roles of TMA and TMAO in early-stage DKD.

In this study, we measured the levels of urine TMAO and its precursors in the T2DM patients with microalbuminuria (Micro). Furthermore, we analyzed the differences in the gut microbiota composition between healthy individual and T2DM patients with Micro to determine the relationship between gut microbiota composition, TMAO levels, and clinical features associated with early-stage DKD.

## 2 Methods

### 2.1 Research participants and sample collection

A total of 67 participants, including 15 healthy controls (HC) and 52 patients with type 2 diabetes mellitus (T2DM), were recruited from the First Affiliated Hospital of Huzhou University. The diagnosis of T2DM was based on specific criteria, including fasting plasma glucose (FBG) ≥ 7.0 mmol/L, two-hour plasma glucose level of oral glucose tolerance test (OGTT) ≥ 11.1 mmol/L, glycosylated hemoglobin (HbA1c%) ≥ 6.5%, and estimated glomerular filtration rate (eGFR) ≥ 60 mL/min/1.73 m^2^. T2DM patients were then further divided into two groups based on the random urinary albumin creatinine ratio (UACR) levels: patients with normoalbuminuria (n=26; Normo; UACR ≤ 30 mg/g) and patients with Micro (n=26; 30 mg/g < UACR ≤ 300 mg/g). The patients group were recruited consecutively from the Endocrinology Department, while age- and gender-matched healthy individuals were recruited from the Health Examination Center. All participants belonged to the Huzhou Han nationality. They were from similar geographic areas and had similar eating habits.

Exclusion criteria included pregnant diabetes patients, patients with type 1 diabetes or diabetes ketoacidosis, patients with other specific types of diabetes, non-diabetic kidney disease, intestinal diseases, usage of antibiotics within the past month, acute/chronic infections, patients with malignant tumors, and any mental state that would restrict the subject from consenting to the study.

The following data was extracted from the hospital’s public clinical database for the included patients: (1) clinical data, including age, gender, and history of diabetes; (2) laboratory indices, including FBG, insulin (INS), C-peptide (C-p), HbA1c%, total cholesterol (TC), triglycerides (TG), low-density lipoprotein (LDL-C), high-density lipoprotein (HDL-C), hemoglobin (Hb), serum creatinine (Scr), blood urea nitrogen (BUN), eGFR (CKD-EPI, ml/min/1.73m2), albumin (ALB), Urine RBC count, UACR. The HC group also underwent various tests, including blood analysis, urine analysis, hepatitis B surface antigen (HBsAg), anti-hepatitis C antibody (HCV), liver and kidney function, and ultrasound Doppler examination. The results of these tests were found to be within the normal range.

Fasting urine and fecal samples from all participants were collected in the morning, stored in ice bags, transferred within 2 hours to the laboratory and frozen at -80°C until further analysis. It is important to note that all subjects followed an omnivorous diet, and none of them reported having any special eating habits.

### 2.2 Quantification of the urine TMAO, TMA, and the precursors concentrations

The concentrations of TMAO, TMA, betaine, choline, L-carnitine and creatinine (Cr) in the urine samples were quantified using stable isotope dilution Ultra performance liquid chromatography tandem mass spectrometry (UPLC-MS/MS, SCIEX 6500 QTRAP+ triple quadrupole mass spectrometer). Briefly, 40 *μ*L of 0.1% formic acid aqueous solution was added to 10 *μ*L sample. Subsequently, 200 *μ*L of extraction solution (0.1% formic acid acetonitrile containing isotopically-labeled internal standard mixture, including TMAO-d9, TMA-d9, betaine-d3, choline-d9, L-carnitine-d9, and Cr-d3 at 10 mmol/L) that had been precooled at -20°C was added to the above mentioned urine-formic acid mixture. The mixture was vortexed, sonicated in an ice-water bath for 15 minutes, and then incubated at -40°C for 1 hour. After centrifugation, an 80 *μ*L supernatant was transferred to an auto-sampler vial, and 1 μL supernatant was injected onto a Waters ACQUITY BEH Amide column (100 × 2.1 mm, 1.7 μm, Waters) at a flow rate of 0.5 mL/min using 77% solvent A (10 mmol/L ammonium formate and 1% formic acid in water) and 23% solvent B (1% formic acid in acetonitrile). Mass spectrometry analysis was conducted in positive multiple reaction monitoring (MRM) mode. The acquired MRM data were processed using SCIEX Analyst Work Station Software (version 1.6.3) and Sciex MultiQuant™ Software (version 3.0.3).

### 2.3 Fecal DNA extraction and 16S ribosomal DNA gene sequencing

Genomic DNA was extracted from fecal samples using the PowerFecal^®^ DNA Isolation Kit (MoBio) according to the manufacturer’s protocol. The quantity of genomic DNA was determined using a TBS-380 fluorometer (Turner BioSystems Inc., Sunnyvale, CA), and high-quality DNA (OD260/280 = 1.8~2.0, >1 μg) was used for further analysis.

The V3-V4 variable regions of the bacterial 16S ribosomal DNA gene (16S rDNA) were amplified by polymerase chain reaction (PCR) using the barcoded primers 338F (ACTCCTACGGGAGGCAGCA) and 806R (GGACTACHVGGGTWTCTAAT). The PCR products were extracted, purified, and sequenced with a high throughput sequencer using the MiSeq platform (Illumina Miseq PE300, USA) according to the manufacturer’s protocol. The above procedures were completed by Shanghai Majorbio Technology Co., Ltd.,Shanghai, China.

### 2.4 Statistical analyses

#### 2.4.1 Descriptive analyses and metabolome analyses

Statistical analyses were performed using SPSS 22.0 (SPSS Inc., Chicago, IL, USA). The data were presented as either mean ± standard deviation (SD) or median (quartile) for continuous variables, and as absolute values and percentages (%) for categorical variables. Independent t-tests or analysis of variance (ANOVA) were used to compare continuous variables among groups, depending on the normal or non-normal distribution characteristics, respectively. Mann-Whitney U test was employed for analyzing the non-normally distributed data. Categorical variables were evaluated using the chi-squared test. Two-tailed P <0.05 was considered statistically significant.

#### 2.4.2 Microbiome analyses

All statistical analyses were conducted using the R software (version 3.6.3). Alpha-diversity (community richness indices: sobs, chao, ace, and community indices: shannon, simpson, bergerparker) and beta-diversity (Bray-Curtis dissimilarity) indices were generated using the vegdist and diversity functions in the vegan R package. One-way ANOVA was used to evaluate the differences in alpha-diversity between groups. Permutational analysis of variance (PERMANOVA) for the Bray-Curtis dissimilarity, implemented in the adonis R function (17), was performed to evaluate the multidimensional centroid differences in beta-diversity among groups. The Kruskal-Wallis H test was employed to evaluate the differential abundance of the genera. Differences in the relative abundances of microbial features were determined using linear discriminant analysis (LDA) effect size (LEfSe). LEfSe and the non-parametric Kruskal-Wallis (KW) sum-rank test were used to analyze the differences in the relative abundance between groups from the phylum to the genus level, and to identify significantly different species. Subsequently, LDA was used to obtain species with a score greater than 2.5. The correlation between biochemical indicators, metabolites, and various microorganisms was calculated using Spearman’s rank correlation coefficient. The results were visualized using the “pheatmap” software package through a heatmap in R.

## 3 Results

### 3.1 Clinical characteristics of all participants

**Figure 1:**
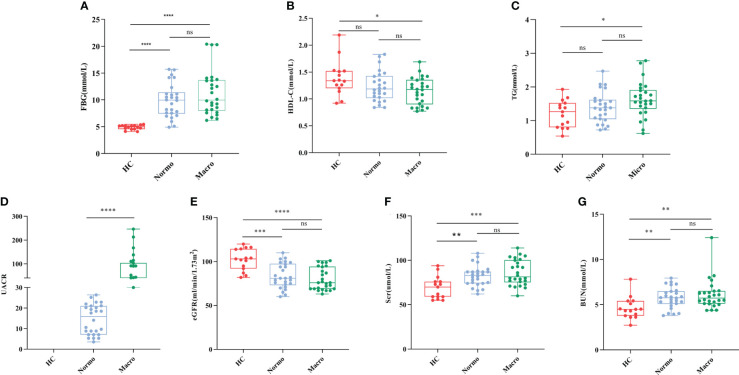
Key biochemical indicators between the three groups. **(A)** FBG, **(B)** HDL-C, **(C)** TG, **(D)** UACR, **(E)** eGFR, **(F)** Scr, and **(G)** BUN. *P <0.05; **P <0.01; ***P <0.001; ****P <0.0001. HC, Healthy Controls; Normo, T2DM patients with normoalbuminuria; Micro, T2DM patients with microalbuminuria.

After quality control, a total of 67 participants were included in this study. The key clinical characteristics at the time of sample collection are presented in **Table 1** and **Figure 1**. Compared to the HC, patients with T2DM (both Normo and Micro) showed significantly increased levels of FBG, Scr, and BUN, as well as significantly reduced levels of eGFR(CKD-EPI, ml/min/1.73m^2^). The UACR of T2DM patients with Micro were significantly different from the T2DM patients with Normo, but the other clinical and laboratory indicators between the 2 groups did not show significant differences. There were no significant differences in age and gender among the three participant groups.

**Table 1:** Key clinical characteristics and laboratory data of the participants.

	HC (n=15)	Normo (n=26)	Micro (n=26)	*P*
Age (yr)	48.73 ± 7.04	53.65 ± 9.22	52.92 ± 9.19	0.1245
Male (%)	8(53.33%)	15(57.69%)	16(61.54%)	0.2679
Diabetes duration(yr)	0.00 ± 0.00	6.89 ± 5.70	8.28 ± 6.3	<0.001
FBG (mmol/L)	4.88 ± 0.42	9.86 ± 3.15^a^	11.28 ± 4.19^b^	<0.001
INS (uU/ml)	NA	6.55(4.85,12.53)	8.9(6.55,15.58)	0.2003
C-p (ng/ml)	NA	1.48 ± 0.69	1.78 ± 0.73	0.1420
HbA1c(%)	NA	9.20 ± 2.58	9.59 ± 2.23	0.5637
TC (mmol/L)	4.77 ± 0.68	4.52 ± 0.75	5.02 ± 0.91	0.0875
TG (mmol/L)	1.21 ± 0.41	1.39 ± 0.46	1.63 ± 0.52^b^	0.0210
LDL-C (mmol/L)	2.57 ± 0.5	2.39 ± 0.63	2.53 ± 0.70	0.6404
HDL-C (mmol/L)	1.39 ± 0.32	1.23 ± 0.28	1.15 ± 0.25^b^	0.0343
Hb (g/L)	142.9 ± 16.07	142.7 ± 15.31	146.4 ± 18.32	0.6876
Scr (μmol/L)	69.2 ± 12.68	82.42 ± 11.61^a^	86.12 ± 14.35^b^	<0.001
BUN(mmol/L)	4.49(3.78,5.41)	5.76(5.07,6.49)^a^	5.72(5.11,6.52)^b^	0.0019
eGFR	103(92,114.4)	81(73.25,97.5)^a^	76(69,94.25)^b^	<0.001
ALB (g/L)	44.2(43.4,46.3)	44.1(41.08,49.4)	47.15(45.93,50.28)	0.0711
Urine RBC count (n/ul)	0(0,0)	0(0,0)	0(0,0.35)	0.7345
UACR (mg/g)	NA	15.84(7.04,21.03)	40.92(34.51,104.1)^c^	<0.001

Categorical variables are expressed as n (%); continuous variables are expressed as median (interquartile range). HC, Healthy Controls; Normo, T2DM patients with normoalbuminuria; Micro, T2DM patients with microalbuminuria. ^a^P ^<^0.05: HC versus Normo; ^b^P ^<^0.05: HC versus Microa; ^c^P ^<^0.05: Normo versus Micro.

### 3.2 Concentrations of TMAO, TMA, and the precursor metabolites in all participants

**Figure 2:**
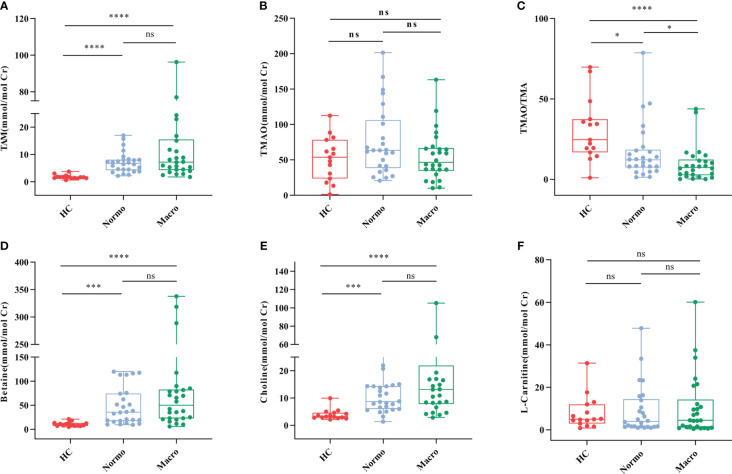
Urine metabolic indicators in the three groups. **(A)** TMA, **(B)** TMAO, **(C)** TMAO/TMA, **(D)** Betaine, **(E)** Choline, and **(F)** L-Carnitine. *P <0.05; ***P <0.001; ****P <0.0001. HC, Healthy Controls; Normo, T2DM patients with normoalbuminuria; Micro, T2DM patients with microalbuminuria.

The concentrations of six urine metabolites, namely TAM, TMAO, choline, betaine, L-carnitine and Cr, were quantified using Stable Nuclide Dilution UPLC-MS/MS. After adjusting for baseline Cr levels, the relative levels of the methylamine metabolites in urine samples from the three groups were estimated and shown in **Table 2** and **Figure 2**. Compared to the HC, Normo and Micro patients exhibited significantly higher levels of TAM, choline and betaine in urine, along with significantly lower TMAO/TMA ratios. Additionally, TMAO levels were slightly increased in the Normo group and slightly decreased in the Micro group compared with the HC group, but the differences between the 3 groups were not statistically significant. The data showed significant differences in TMAO/TMA, TMA and its precursor metabolites among the three groups (p < 0.001).

**Table 2:** Concentrations of TAM, TMAO, choline, betaine, and L-carnitine in the participants.

mmol/mol Cr in Urine	HC (n=15)	Normo (n=26)	Micro (n=26)	*P*
TMA	1.68(1.33,1.83)	6.75(4.27,8.08)^a^	7.23(4.31,15.52)^b^	<0.001
TMAO	53.64(23.69,78.35)	63.53(39.97,106)	46.48(34.59,66.73)	0.1665
TMAO/TMA(mol/mol)	24.61(16.76,37.39)	12.12(7.24,18.48)^a^	7.39(2.57,12.28)^b,c^	<0.001
Choline	3.19(2.79,4.6)	8.72(5.96,14.33)^a^	13.18(7.84,21.91)^b^	<0.001
Betaine	9.13(7.29,13.25)	35.61(17.23,74.5)^a^	50.11(22.62,82.64)^b^	<0.001
L-Carnitine	4.95(2.9,12)	3.95(1.51,14.41)	4.46(1.26,14.24)	0.9204

HC, Healthy Controls; Normo, T2DM patients with normoalbuminuria; Micro, T2DM patients with microalbuminuria. ^a^P ^<^0.05: HC versus Normo; ^b^P ^<^0.05: HC versus Microa; ^c^P ^<^0.05: Normo versus Micro.

### 3.3 Gut microbiota diversity among healthy controls, T2DM with normoalbuminuria, and T2DM with microalbuminuria

Gut microbiome analysis was conducted on 67 samples from the HC, Normo, and Micro groups. A total of 3,738,982 high-quality 16S rDNA reads were obtained, with a median reading count of 54,394 (ranging from 40,805 to 73,942). Subsequently, clustering analysis of sequences with a similarity greater than 97%, 1 domain, 1 kingdom, 11 phyla, 17 classes, 24 orders, 49 families, 177 genera, 350 species, and 523 operational taxonomic units (OTUs) were obtained.

**Figure 3:**
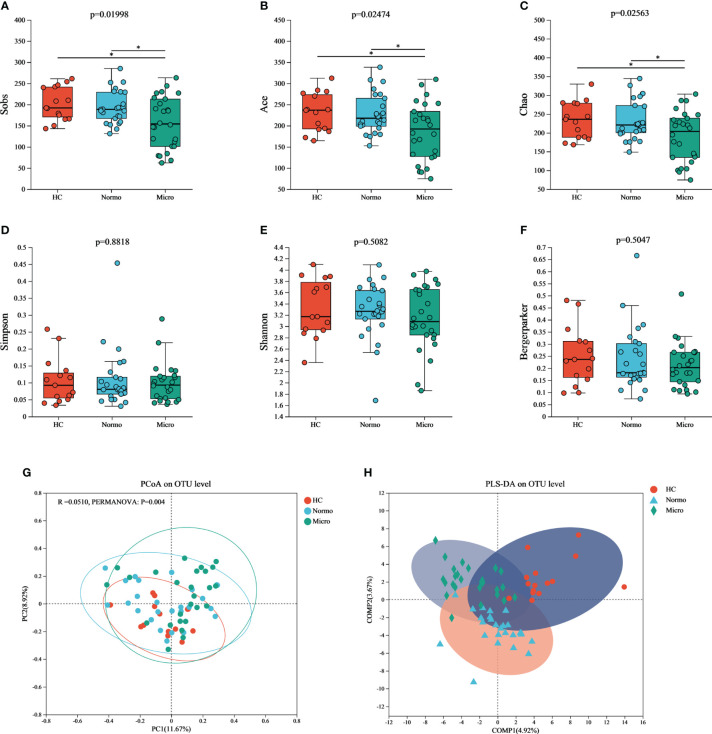
α and β diversity of gut microflora among three groups at OTU level. Community richness indices: **(A)** Sobs, **(B)** Ace, and **(C)** Chao; Community diversity indices: **(D)** Simpson, **(E)** Shannon, and **(F)** Bergermaker; β diversity indices: **(G)** PCoA, and **(H)** PLS-DA. *P <0.05; HC, Healthy Controls; Normo, T2DM patients with normoalbuminuria; Micro, T2DM patients with microalbuminuria.

To evaluate the α diversity of the gut microflora between the 3 groups, various indices including Sobs, Chao, ACE (community richness), Shannon, Simpson, and Berger-Parker (community diversity) were calculated based on the OTU profiles. The α diversity results (**Figure 3**) demonstrated that Sobs, Chao, and ACE indices were significant reduced in the Micro group compared with the HC group and Normo group. However, there were no singnificant diffrerences in Simpson, Shannon, and Berger-Parker indices in the 3 groups. These findings suggested that the gut microflora richness was decreased significantly in the Micro group compared to the HC and Normo groups, while the diversity remained unchanged. No statistical difference in α diversity was observed between the HC and Normo groups.

Principal Coordinate Analysis (PCoA) based on Jaccard dissimilarity and Bray-Curtis dissimilarity was performed to assess the β diversity and identify significant differences in microbial community structure. **Figure 3G** showed the separation of microbiota composition at the OTU level among patients with HC, Normo, and Micro (PERMANOVA, p = 0.004). Furthermore, partial least squares discriminant analysis (PLS-DA, **Figure 3H**) demonstrated clear distinction and clustering of HC, Normo and Micro samples, indicating a significant differences in intestinal flora composition.

### 3.4 Gut microbiota composition among healthy controls, T2DM with normoalbuminuria, and T2DM with microalbuminuria

**Figure 4:**
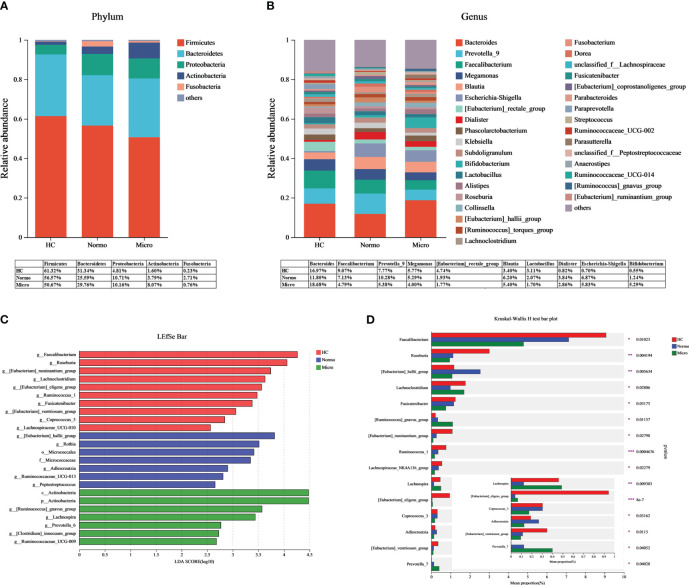
Gut microbiota composition between HC, Normo and Micro. **(A)** Gut microbiota composition at the phylum level. **(B)** Gut microbiota composition at the genus level. Bacteria that took up <1% of the microbiota were labeled together as “others”. **(C)** Bacterial taxa differences among the three groups using LEfSe analysis, Only taxa meeting the LDA significance thresholds > 2.5 were shown. **(D)** Top 15 differentially bacterial genera in the 3 groups. The comparison of the mean abundance for the bacterial genera between the three groups was based on Kruskal-Wallis H test. *P <0.05, **P <0.01, ***P <0.001; HC, Healthy Controls; Normo, T2DM patients with normoalbuminuria; Micro, T2DM patients with microalbuminuria.

Bacteria with relative abundances greater than 1% at the phylum and genus levels were compared between the HC, Normo, and Micro groups. At the phylum level (**Figure 4A**), Firmicutes, Bacteroidetes, Proteobacteria, Actinobacteria and Fusobacteria accounted for 99% of the bacteria. Firmicutes was the dominant phylum, representing over 50% in each group. At the genus level (**Figure 4B**), among the bacteria with relative abundances greater than 3%, Bacteroides, Faecalibacterium, Prevotella_9, Megamonas and Blautia were common to allthe three groups. The HC group additionally included [Eubacterium]_rectale_group, Lactobacillus, Klebsiella and Roseburia; the Normo group included Escherichia-Shigella and Dialister; the Micro group featured Escherichia-Shigella and Bifidobacterium.

LDA-LEfSe analysis was performed to determine significant differences in the bacterial abundances between the 3 groups and identify the bacterial genera that are associated with the Micro group. Based on the LDA score threshold of above 2.5, a total of 24 distinguishing taxa with differential abundances were labeled between the three groups (**Figure 4C**), ranging from phylum to genus level. At the genus level, Faecalibacterium, Roseburia, [Eubacterium]_ruminantium_group, Lachnoclostridium, [Eubacterium]_eligens_group, Ruminococcus_1, Fusicatenibacter, [Eubacterium]_ventriosum_group, Coprococcus_3 and Lachnospiraceae_UCG-010 were significantly enriched in the HC group. [Eubacterium]_hallii_group, Rothia, Adlercreutzia, Ruminococcaceae_UCG-013 and Peptostreptococcus were significantly higher in the Normo group. The Micro group showed an overrepresentation of [Ruminococcus]_gnavus_group, Lachnospira, Prevotella_6, [Clostridium]_innocuum_group and Ruminococcaceae_UCG-009. The Kruskal-Wallis H test was then used to analyze the top 15 genera with the most significant differences in average abundance between the three groups (**Figure 4D**). The results revealed decreased abundance of eight bacterial genera (Faecalibacterium, Roseburia, Fusicatenibacter, [Eubacterium]_ruminantium_group, Lachnospiraceae_NK4A136_group, Ruminococcus_1, [Eubacterium]_ventriosum_group and Coprococcus_3) and increased abundance of two bacterial genera ([Ruminococcus]_gnavus_group and Prevotella_7) during the HC-Normo-Micro transition. This suggested the potential relevance of these 10 bacterial genera in the development and progression of T2DM.

### 3.5 Correlation analysis of gut microbiota, clinical characteristics, and metabolites in healthy controls, T2DM with normoalbuminuria, and T2DM with microalbuminuria

**Figure 5:**
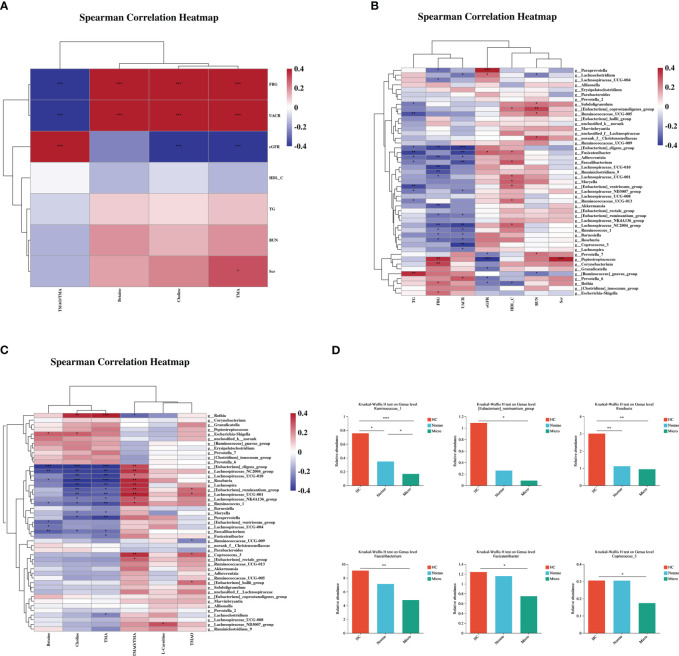
Correlations of gut microbiota composition, clinical characteristics, and metabolites between the three groups. **(A)** Correlation of differentially gut microbiota and differential clinical features beween the three groups. **(B)** Correlation of differential metabolites and differential clinical features between the three groups. **(C)** Correlation of differential metabolites and differentially gut microbiota between the three groups. **(D)** Key bacteria associated with clinical features or metabolism in the three groups. *P <0.05, **P <0.01, ***P <0.001; HC, Healthy Controls; Normo, type 2 diabetes patients with normoalbuminuria; Micro, type 2 diabetes patients with microalbuminuria.

Spearman correlation analysis was performed to assess the relationships between the various differentially bacterial genera(P<0.1), clinical indices and metabolite levels among the three groups (**Figure 5**). Initially, the metabolites associated with renal function were analyzed. TMA, choline and betaine showed positive correlation with UACR and FBG, while TMAO/TMA ratio showed an inverse relationship. TMA also displayed a positive correlation with Scr (**Figure 5A**). Next, the differentially bacteria associated with renal function were examined, UACR showed a strong negative correlation with [Eubacterium]_eligens_group, Fusicatenibacter, Faecalibacterium, Lachnospiraceae_NC2004_group, and Coprococcus_3, and a positive correlation with Prevotella_6. Peptostreptococcus demonstrated a strong positive correlation with Scr (**Figure 5B**). Additionally, the correlation between the metabolites and the differentially bacteria was analyzed. TMA and choline levels showed significant negative correlations with 9 bacterial genera ([Eubacterium]_eligens_group, Roseburia, Lachnospira, Lachnospiraceae_NC2004_group, Lachnospiraceae_UCG-010, Ruminococcus_1, [Eubacterium]_ruminantium_group, Lachnospiraceae_UCG-001 and Paraprevotella) and a significant positive correlation with Rothia. The TMAO/TMA ratio demonstrated a strong positive correlation with Roseburia, [Eubacterium]_eligens_group, Lachnospiraceae_NC2004_group, Lachnospira, [Eubacterium_ruminantium_group, Lachnospiraceae_UCG-010 and Coprococcus_3. Betaine showed a strong negative correlation with [Eubacterium]_eligens_group, Lachnospiraceae_NC2004_group, and Faecalibacterium (**Figure 5C**).

Through the correlation analysis, it was observed that Ruminococcus_1, [Eubacterium]_ruminantium_group, Lachnospiraceae_NC2004_group, Roseburia, [Eubacterium]_eligens_group, Lachnoclostridum, Moryella, Faecalibacterium and Fusicatenibacter were not only significantly correlated with TMA but also exhibited significant correlations with UACR. Furthermore, Coprococcus_3, Ruminococcus_1, [Eubacterium]_ruminantium_group, Lachnospiraceae_NC2004_group, Roseburia and [Eubacterium]_eligens_group showed a positive correlation with TMAO/TMA ratio and a negative correlation with UACR. Lachnospiraceae_NC2004_group, Roseburia, Ruminococcus_1, [Eubacterium]_eligens_group and Faecalibacterium demonstrated significant correlations with betaine and UACR. Based on the findings from **Figures 2** and **4**, we performed detailed analysis of the differences between the 3 groups and observed that the relative abundances of six bacterial genera, including [Eubacterium]_ruminantium_group, Ruminococcus_1, Roseburia, Faecalibacterium, Fusicatenibacter and Coprococcus_3 were significantly different among the 3 groups (**Figure 5D**).

## 4 Discussion

DKD is a severe microvascular complication of diabetes influenced by various factors, including blood pressure, hyperglycemia, body mass index (BMI), and others. Recent studies have shown that gut dysbiosis is associated with T2DM. TMAO is the independent risk factor of DKD. Therefore, in this study, we analyzed urine metabolites, and the composition of the gut microbiota to determine whether the relationship between gut dysbiosis and the early renal complications in patients with T2DM. The results demonstrated dysbiosis of the gut microbiota and increased levels of TMA and its precursors in the urine of T2DM patients with Micro compared to HC and T2DM patients with Normo. Furthermore, six bacterial genera that showed significant differences in the relative abundances between the three groups, and these differentially bacteria were found to be associated with elevated UACR and metabolic levels of TMAO/TMA, TMA, and its precursors.

Previous studies have reported that TMAO, a gut microbiota metabolite, is a risk factor for CVD and stroke (18, 19) and is also linked with CKD progression and all-cause mortality (20). However, our research showed no significant differences in urine TMAO levels between the three groups. This suggested that urine TMAO levels remain relatively stable during the early stages of DKD and are consistent with the findings by Caroline C. Pelletier et al. (21). Further investigation is needed to determine the predictive value of plasma or urinary TMAO levels for early DKD. Interestingly, similar to the previous studies, the levels of TMA and its precursors (choline and betaine) increased sequentially among the three groups, while TMAO/TMA ratio decreased sequentially, and these changes were associated with changes in the levels of the renal function biomarkers (22). TMA has long been recognized as a uremic toxin (23), and its accumulation is associated with the increased risk of CVD (18). Therefore, regular monitoring of the TMA levels in the early stage of DKD may be warranted.

T2DM patients with Micro exhibited significantly reduced bacterial richness. These findings were in alignment with a recent systematic review and meta-analysis that reported gut microbiota dysbiosis in DKD (24). Therefore, these data demonstrated the occurrence of gut dysbiosis in the early stages of DKD. Short-chain fatty acid (SCFA), including Eubacterium, are key bacterial metabolites that can influence various physiological and pathological processes such as energy metabolism, blood sugar control, intestinal immunity, and so on (25). Butyrate can improve angiotensin II-mediated renal injury by affecting urinary protein production, glomerulosclerosis, renal fibrosis, and inflammation (26). Faecalibacterium, Fusicatenibacter, Lachnospiraceae_NK4A136_group, Roseburia, Ruminococcus_1 and Coprococcus_3 are beneficial butyrate-producing bacteria, which have been reported to be depleted in patients with DKD, IgA nephropathy, obesity, cardiovascular and other diseases (27–30). The [Eubacterium]_ventriosum_group was noticeably decreased in the gut of the T2DM rat model and was negatively associated with the expression of T2DM-related biomarkers (31). [Ruminococcus]_gnavus_group as a harmful bacteriumis, enriched in individuals with prediabetes and insulin resistance (32). Prevotella is generally associated with a healthy plant-based diet and acts as a “probiotic” in the human body. However, recent human research has demonstrated that increased abundance of Prevotella is associated with local and systemic diseases, including periodontal disease, rheumatoid arthritis, and metabolic disorders (33, 34). In summary, the research results indicate that dysbiosis of the gut microbiome in the early stage of DKD are characterized by depletion of SCFA-producing bacteria and enrichment of harmful bacteria.

In addition, our findings indicate a strong correlation between SCFA-producing bacterial genera and Eubacterium with the levels of microbiota-derived nephrotoxins, such as TMA and TMAO/TMA ratio in urine. This suggested that SCFA-producing genera and Eubacterium may play a crucial role in suppressing the synthesis of TMA. Previous studies have found that in CKD, two SCFA-producing genera (Pseudobutyrivibrio and Dialister) were inversely correlated with the levels of circulating indoxy sulfonate (35). Furthermore, treatment of CKD mice with Faecalibacterium praussnitzii ameliorated renal dysfunction by attenuating renal inflammation, increasing butyrate levels, and markedly reducing the circulating levels of uremic toxins such as p-cresol sulfate, TMAO, and guanosuccinate (36). Colonization of TMA-producing bacteria alone did not increase TMA levels in the cecum or TMAO leves in the serum. However, when TMA-producing bacteria were colonized along with other intestinal bacterial strains, a significant increase in the relative abundance of TMA producing bacteria in the small gut and the level of TMAO in the serum was observed (37). These findings suggested that the interactions between gut bacteria, particularly SCFA-producing genera and Eubacterium, may influence the production of TMA. However, additional research is required to gain a deeper understanding of the specific mechanisms by which the SCFA-producing genera and Eubacterium influence TMA production.

### 4.1 Limitations

It is important to acknowledge several limitations of this study. Firstly, the sample size in this study was small. Therefore, larger multicenter studies are required to elucidate the true relationship between gut dysbiosis and the development of DKD. Nonetheless, all participants in this study were residents of Huzhou City, Zhejiang Province, and showed relatively centralized and consistent characteristics and living habits. Secondly, the relative abundances of gut microbiota were based on 16S rRNA gene sequencing. Further analysis based on gut metagenomes is needed to provide more bacterial information and functional genes. Thirdly, the cross-sectional design of our study limits any conclusions about the directionality or causality of the identified microbiota and the metabolite features.

## 5 Conclusions

In conclusion, the increased TMA metabolism was associated with the imbalance of gut microbiota during the inital stage of DKD. This research provides relevant information that can be used to develop early prevention strategies for DKD. These findings demonstrated the importance of the communication between the gut microbiota and kidney function for the management of DKD. However, further clinical and animal experiments are necessary for confirming the relationship between rebalancing the gut microbiota composition and the levels of the microbial metabolites, including SCFAs and uremia toxins such as TMA.

## Data availability statement

The data presented in the study are deposited in the NCBI repository, accession number PRJNA1000711.

## Ethics statement

The studies involving humans were reviewed and approved by the Ethics Committee of the Medical Center of the First Affiliated Hospital of Huzhou University. The ethical approval code was 2018KY043. The participants provided their written informed consent to participate in this study. The studies were conducted in accordance with the local legislation and institutional requirements.

## Author contributions

LH: Funding acquisition, Methodology, Software, Writing – original draft, Writing – review & editing. HL: Software, Writing – original draft, Writing – review & editing. MZ: Funding acquisition, Investigation, Writing – review & editing. YL: Methodology, Writing – review & editing. LR: Data curation, Funding acquisition, Investigation, Writing – review & editing. JH: Data curation, Investigation, Writing – review & editing. XW: Funding acquisition, Investigation, Project administration, Writing – review & editing.
